# Cherry Pectoral

**Published:** 1854-02

**Authors:** 


The following is understood to be the formula of Ayer's Cherry Pectoral:

| | |
|---|---|
| ℞.—Morph. Acetas, - - - - - | grains iv. |
| Tinct. Sanguinariæ, - - - - - - | drams ij. |
| Wine of Antimony, - - - - - | " iij. |
| Wine of Ipecac, - - - - - - | " iij. |
| Syrup of Wild Cherry Bark, - - - | ounces iij. |

